# Clinical Outcomes of Early vs. Late Tracheostomy in Ventilated COVID-19 Patients

**DOI:** 10.7759/cureus.63757

**Published:** 2024-07-03

**Authors:** Heba Alkoheji, Lana Alabbasi, Mubarak S Aldoseri, Khalifa Abdulrahman Yusuf, Mai Nasser, Jalal Alkhan, Manaf Alqahtani, Mohamed Alshehabi

**Affiliations:** 1 Otolaryngology, Royal Medical Services Military Hospital, Riffa, BHR; 2 Internal Medicine, King Hamad University Hospital, Busaiteen, BHR; 3 Anaesthesia, Royal Medical Services Military Hospital, Riffa, BHR; 4 Internal Medicine, Royal Medical Services Military Hospital, Riffa, BHR; 5 Paediatric Otolaryngology, Royal Medical Services Military Hospital, Riffa, BHR; 6 Infectious Disease, Royal Medical Services Military Hospital, Riffa, BHR; 7 Otolaryngology - Head and Neck Surgery, Royal Medical Services Military Hospital, Riffa, BHR

**Keywords:** bacterial infections, world pandemic, covid-19, late tracheostomy, early tracheostomy, tracheostomy

## Abstract

Background

The coronavirus disease 2019 (COVID-19) global pandemic prompted a significant use of intensive care resources for managing hypoxic respiratory failure. A substantial portion of these patients required mechanical ventilation. While intubation is common, its impact on mortality improvement has been questionable. Tracheostomies have become crucial for patients needing prolonged ventilation. However, tracheostomies also risk infections, ranging from early-stage mild cellulitis to later-stage nosocomial pneumonia. Our study evaluates the incidence of bacterial infections in COVID-19 patients who underwent tracheostomy early (within 14 days) versus late (more than 14 days after initiation of mechanical ventilation) during their stay in the intensive care unit (ICU).

Methods

We conducted a retrospective single-center study at Royal Medical Services Military Hospital. The study included COVID-19 patients who underwent tracheostomy and were admitted to the ICU from March 2020 to March 2022. We analyzed the incidence of ventilator-associated pneumonia, the timing of weaning from mechanical ventilation, and outcomes between early and late tracheostomized patients. Analyzed variables included demographics, comorbidities, use of steroids, tocilizumab, inflammation parameters, tracheostomy timing, incidence of bacterial infections, complications, and outcomes.

Results

The study comprised 36 patients. We found no statistically significant difference in the incidence of bacterial infections between the early and late tracheostomy groups (P>0.05). Complications and overall outcomes did not show significant statistical associations. Inotropes use was more frequent in the late tracheostomy group (P=0.122). In contrast, continuous renal replacement therapy was higher in the early tracheostomy group, showing no significant association (P>0.05). Mortality was higher in the early tracheostomy group, with nine deaths compared to seven in the late tracheostomy group. Interestingly, infection with Acinetobacter baumannii was associated with a statistically significant lower mortality rate, with 75% survival following tracheostomy.

Conclusions

Findings suggest that tracheostomy timing does not significantly impact the incidence of bacterial pneumonia or other complications, such as the use of inotropes, continuous renal replacement therapy, or mortality rates. These results support the use of personalized decision-making while conducting tracheostomies. Further research is necessary to determine the impacts of tracheostomy timing on patient outcomes more definitively.

## Introduction

The coronavirus disease 2019 (COVID-19) global pandemic significantly impacted healthcare, particularly critical care [1]. Approximately 17% to 35% of hospitalized COVID-19 patients require admission to an intensive care unit (ICU) due to hypoxic respiratory failure. Of these, over 75% need supplemental oxygen, and 3% to 15% require mechanical ventilation. However, an association between mortality and mechanical ventilation has been recently investigated, where in a study of 52 critically ill COVID-19 patients, 30 of the 47 mechanically ventilated patients (81%) were dead by 28 days. Moreover, by the end of the study, no intubated patients were extubated and alive [2].

Tracheostomy, a mainstay in modern critical care, benefits patients needing prolonged ventilation. It allows for the reduction or cessation of sedation and facilitates the removal of the trans-laryngeal tube, aiding in laryngeal rehabilitation. Tracheostomy also provides a stable interface for various ventilatory supports without needing re-sedation or tracheal reintubation [1]. In COVID-19 patients, tracheostomy not only improves survival rates but also aids in weaning from ventilatory support and alleviates the strain on critical care resources [1].

Tracheostomy, compared to endotracheal intubation, generally reduces the work of breathing, lowers sedation needs, facilitates weaning, and improves communication and swallowing [3]. However, it also presents risks. Early complications within 10 days can include hemorrhage, obstruction, accidental decannulation, pneumothorax, and infection. Late complications may involve stenosis, tracheomalacia, fistula formation, dysphagia, dysphonia, and aspiration pneumonia. It remains unclear whether tracheostomy reduces or increases the incidence of nosocomial pneumonia compared to endotracheal intubation, as studies present conflicting data. In general, infections are a prevalent postoperative complication. Early infections typically include mild cellulitis at the stoma site or tracheitis, while more severe bacterial infections such as abscesses and mediastinitis may also occur and require antibiotic treatment. Late complications often involve aspiration and nosocomial pneumonia [4].

The timing of tracheostomy in COVID-19 patients has sparked debate. Decision-making has been influenced by considerations of early versus late tracheostomy, assumptions about the infectivity timeline of the virus, and concerns about the risks to surgeons due to potential aerosol generation during the procedure [5]. Optimal timing remains controversial. While delayed tracheostomy minimizes risk to healthcare staff, early tracheostomy can reduce laryngeal damage and dysfunction from prolonged intubation, decrease sedative use, and enhance pulmonary hygiene through better secretion management [1].

Despite numerous studies, the optimal timing for tracheostomy is still debated, with previous meta-analyses showing varying effects on clinical outcomes [6].

This study aims to determine the incidence of bacterial infections in early versus late tracheostomized COVID-19 patients admitted to the ICU at Royal Medical Services Military Hospital. In this study, an early tracheostomy is defined as one performed within 14 days of starting mechanical ventilation, whereas a late tracheostomy occurs after this period.

## Materials and methods

In this retrospective single-center study conducted at Royal Medical Services Military Hospital, COVID-19 patients admitted to the ICU from March 2020 to March 2022 who underwent tracheostomy were included. Approval was obtained from the institutional research and ethical board and the national COVID-19 team’s ethical board (Approval No. 2023-673). Data were collected retrospectively from participants’ electronic medical records. The variables included demographics (age, sex, body mass index, nationality), comorbidities, use of steroids, use of tocilizumab, inflammation parameters (white blood cell count, C-reactive protein, procalcitonin levels), timing of tracheostomy, incidence of bacterial infections, complications, and outcomes.

The primary objective was to compare the incidence of ventilator-associated pneumonia in patients who underwent early (within 14 days) versus late (more than 14 days after initiation of mechanical ventilation) tracheostomy. The secondary objective was to assess the time required to wean from mechanical ventilation between the two groups.

Continuous variables, which were not normally distributed as determined by the Shapiro-Wilk normality test, were summarized as mean (standard deviation) and median (first and third quartiles). Categorical variables were summarized as frequencies and percentages. We divided patients into two groups: early tracheostomy (less than 14 days) and late tracheostomy (14 days or more). Baseline characteristics, complications, and outcomes were compared between the two groups using the Mann-Whitney U test for continuous variables and the Chi-square and Fisher’s Exact test for categorical variables. We used IBM SPSS Statistics for Windows, Version 26.0 (IBM Corp., Armonk, NY) for data analysis. A P-value of less than 0.05 was considered statistically significant.

In the present study, no exclusion criteria were used.

## Results

This study included 36 patients. Their baseline characteristics are summarized in Table 1. Analysis revealed no statistically significant differences in baseline characteristics such as age, comorbidities, or steroids use and tocilizumab between the early and late tracheostomy groups.

**Table 1:** Comparison of early and late tracheostomies' baseline characteristics *Benign prostatic hyperplasia, ex-smoker, gout and obesity, morbid obesity and anxiety, multiple myeloma and gastritis, peripheral vascular disease, and sickle cell disease. The P-value was calculated using the Mann-Whitney U test, Chi-square test, and Fisher’s exact test, as appropriate. CKD: Chronic kidney disease; CRP: C-reactive protein; CVA: Cardiovascular accident; IHD: Ischemic heart disease; IQ: Interquartile range; PCT: Procalcitonin; SD: Standard deviation; WBC: White blood cell

Variable		Overall (N=36)	Early Tracheostomy (N=21)	Late Tracheostomy (N=15)	P-Value
Age in years	Mean (SD)	57.25 (12.65)	58 (12)	56 (14)	0.724
	Median (IQR)	61 (52.25, 66.75)	62 (55, 66)	56 (50, 67)	
Comorbidities	Diabetes	15 (41.7)	11 (52.4)	4 (26.7)	0.123
	Hypertension	19 (52.8)	13 (61.9)	6 (40.0)	0.194
	Dyslipidemia	9 (25.0)	7 (33.3)	2 (13.3)	0.252
	IHD	1 (2.8)	0 (0.0)	1 (6.7)	0.417
	CVA	2 (5.6)	1 (4.8)	1 (6.7)	0.806
	CKD	3 (8.3)	2 (9.5)	1 (6.7)	> 0.05
	Other*	7 (19.4)	3 (14.3)	4 (26.7)	0.580
Use of steroids	Yes	35 (97.2)	20 (95.2)	15 (100)	> 0.05
	No	1 (2.8)	1 (4.8)	0 (0.0)	
Use of tocilizumab	Yes	10 (27.8)	5 (23.8)	5 (33.3)	0.709
	No	26 (72.2)	16 (76.2)	10 (66.7)	
WBC	Mean (SD)	11.91 (5.7)	12.33 (5.17)	11.33 (6.52)	0.312
	Median (IQR)	10.47 (8.15, 14.15)	11.07 (9.38, 14.23)	9.14 (6.37, 13.9)	
PCT	Mean (SD)	2.54 (5.87)	2.90 (6.67)	2.04 (4.72)	0.211
	Median (IQR)	0.47 (0.25, 1.72)	0.50 (0.29, 1.90)	0.36 (0.19, 1.17)	
CRP	Mean (SD)	135.2 (116.78)	130.75(120.51)	141.50 (115.21)	0.665
	Median (IQR)	98.02 (32.85, 227.16)	97.04 (28, 212.62)	99 (48.37, 259.82)	

The frequency and association of different bacteria between early and late tracheostomies are presented in Table 2. There was no statistically significant association in the type of bacteria between the two groups. It suggests that the timing of tracheostomy does not significantly influence bacterial infection type.

**Table 2:** Frequency and association of bacteria between early and late tracheostomies The P-value was calculated using the Chi-square test and Fisher’s exact test, as appropriate. Some patients had more than one type of bacteria. MRSA: Methicillin-resistant *Staphylococcus aureus*

Type of Bacteria	Overall (N, %)	Early Tracheostomy (N=21), n (%)	Late Tracheostomy (N=15), n (%)	P-Value
Acinetobacter baumannii	20 (55.6)	11 (52.4)	9 (60.0)	0.650
Pseudomonas aeruginosa	12 (33.3)	6 (28.6)	6 (40.0)	0.473
Stenotrophomonas maltophilia	7 (19.4)	2 (9.5)	5 (33.3)	0.103
Klebsiella pneumonia	12 (33.3)	7 (33.3)	5 (33.3)	> 0.05
Escherichia coli	5 (13.9)	3 (14.3)	2 (13.3)	> 0.05
Candida albicans	3 (8.3)	1 (4.8)	2 (13.3)	0.559
Candida glabrata	1 (2.8)	1 (4.8)	0 (0.0)	> 0.05
MRSA	1 (2.8)	1 (4.8)	0 (0.0)	> 0.05
Serratia marcescens	1 (2.8)	1 (4.8)	0 (0.0)	> 0.05

Complications and outcomes related to tracheostomy timing are detailed in Table 3. There were no significant associations between tracheostomy timing and complications such as the use of inotropes, continuous renal replacement therapy, days spent in tracheostomy, or mortality rates.

**Table 3:** Comparison of early and late tracheostomies' complications and outcomes The P-value was calculated using the Mann-Whitney U test, Chi-square test, and Fisher’s exact test, as appropriate. CRRT: Continuous renal replacement therapy; ICU: Intensive care unit; IQR: Interquartile range; SD: Standard deviation

Variable		Overall (N=36), n (%)	Early Tracheostomy (N=21), n (%)	Late Tracheostomy (N=15), n (%)	P-value
Inotropes	Yes	9 (25.0)	3 (14.3)	6 (40.0)	0.122
	No	27 (75.0)	18 (85.7)	9 (60.0)	
CRRT	Yes	8 (22.2)	5 (23.8)	3 (20.0)	> 0.05
	No	28 (77.8)	16 (76.2)	12 (80.0)	
Days in Tracheostomy in ICU	Mean (SD)	11 (9)	10 (8)	12 (11)	0.822
	Median (IQR)	7 (5, 16)	7 (5, 14)	7 (5, 17)	
Outcome	Alive	20 (55.6)	12 (57.1)	8 (53.3)	0.821
	Dead	16 (44.4)	9 (42.9)	7 (46.7)	

Table 4 presents the relationship between the type of bacteria and mortality rates. Interestingly, infection with *Acinetobacter baumannii* showed a statistically significant association with a lower mortality rate, with 75% of affected patients surviving post-tracheostomy. It contrasts with other bacteria such as *Pseudomonas aeruginosa*, *Stenotrophomonas maltophilia*, and *Klebsiella pneumonia*, which did not show significant differences in survival rates.

**Table 4:** Frequency and association of bacteria with mortality The P-value was calculated using the Chi-square test and Fisher’s exact test, as appropriate. Some patients had more than one type of bacteria. MRSA: Methicillin-resistant *Staphylococcus aureus*

Type of Bacteria	Overall, n (%)	Dead, n (%)	Alive, n (%)	P-value
Acinetobacter baumannii	20 (55.6)	5 (25.0)	15 (75.0)	0.009
Pseudomonas aeruginosa	12 (33.3)	4 (33.3)	8 (66.7)	0.343
Stenotrophomonas maltophilia	7 (19.4)	4 (57.1)	3 (42.9)	0.675
Klebsiella pneumonia	12 (33.3)	4 (33.3)	8 (66.7)	0.343
Escherichia coli	5 (13.9)	2 (40.0)	3 (60.0)	> 0.05
Candida albicans	3 (8.3)	1 (33.3)	2 (66.7)	> 0.05
Candida glabrata	1 (2.8)	1 (100)	0 (0.0)	0.444
MRSA	1 (2.8)	0 (0.0)	1 (100)	> 0.05
Serratia marcescens	1 (2.8)	1 (100)	0 (0.0)	0.444

**Figure 1:**
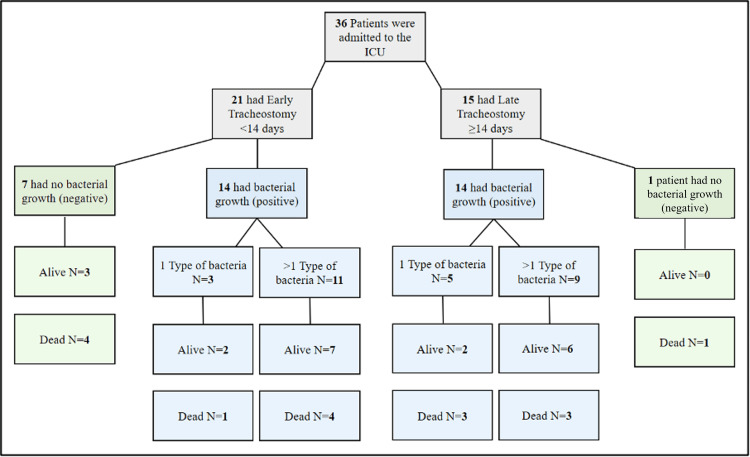
Flow chart of patient mortality following early and late tracheostomies ICU: Intensive care unit

Mortality trends following early and late tracheostomy, differentiated by bacterial infection, are depicted in Figure 1. The findings indicate that the type of bacteria may influence survival outcomes, which warrants further investigation into tracheostomy's clinical management and timing in ICU settings.

## Discussion

The association between tracheostomy timing and clinical outcomes in COVID-19 patients requiring ventilation continues to be debated. In this study, it was found that bacterial infections occurred in both groups; however, there was no statistically significant association between the timing of the procedure and complications. Notably, 56% of identified bacterial infections were due to *A. baumannii *and 33% to *P. aeruginosa*. Although mortality was higher in the late tracheostomy group, these results were not statistically significant. However, the type of bacterial infection correlated with different patient outcomes, where *A. baumannii* was significantly associated with mortality, with 25% of infected patients dying post-tracheostomy.

These findings align with a systematic review by Andriolo et al. [7], which included eight studies assessing the effects of early versus late tracheostomy in critically ill patients. Although early tracheostomy was associated with a lower mortality rate, no definitive evidence suggested a decreased risk of pneumonia. Furthermore, Hosokawa et al. [8] reported no difference in mortality or incidence of ventilator-associated pneumonia between early and late tracheostomy, although early tracheostomy was linked to a shorter duration of sedation and more frequent procedural needs. The duration considered for tracheostomy in these studies was 10 days, compared to the 14 days in this study. It is important to note that both studies were conducted on patients without COVID-19.

The prevalence of *A. baumannii *in tracheostomized patients was highlighted in research by Lipovy et al., where 45.6% of pediatric patients undergoing surgical tracheostomy were most commonly infected with this bacterium, leading to lower respiratory tract infections [9]. However, no significant difference between early and late tracheostomy regarding the gram-positive/gram-negative ratio was observed. Similar findings were reported by Hamidi and Kescioglu, where A. baumannii was present in 49% of patients with late-onset ventilator-associated pneumonia, but it showed no significant impact on survival rates [10].

Conversely, Chorath et al. found that early tracheostomy was associated with a lower incidence of ventilator-associated pneumonia in critically ill patients [6]. Studies by Rumbak et al. [11] and Zheng et al. [12] also indicated a lower risk of pneumonia in the early tracheostomy group, with tracheotomy performed within 48 hours or on day three. A 2017 meta-analysis found that early tracheostomy was associated with a lower risk of pneumonia in trauma patients, using a seven-day cut-off, compared to the 14 days used in our study [13]. It was further supported by Khalili et al. [14], who observed lower ventilator-associated pneumonia rates in the early tracheostomy group among patients with traumatic brain injury. All studies included did not investigate the association with COVID-19.

For instance, there is conflicting evidence regarding the impact of tracheostomy timing on the incidence of secondary bacterial infections in COVID-19 patients. McGrath et al. [15] suggest that decision-making regarding tracheostomy during the COVID-19 pandemic primarily adheres to existing standards of practice, with a weak evidence base for specific timing in critically ill patients.

A study by Ji et al. revealed that early tracheostomy in COVID-19 patients significantly reduced the duration of invasive mechanical ventilation without affecting mortality rates [16].

Battaglini et al. [17] conducted a multicenter study on tracheostomy timing and outcome in severe COVID-19 patients. They found no significant difference in survival between critically ill COVID-19 patients who received tracheostomy before versus after day 15. However, Mishra et al. [18] reported outcomes of tracheostomy in COVID-19 patients. They found promising outcomes for COVID-19 patients who underwent early tracheostomy defined as within 10 days, with higher rates of ventilator liberation and decannulation.

It is in agreement with the multinational cohort study done by Shreckengost et al. [19] in which early tracheostomy (defined as within 14 days of intubation) has reduced dependency on ventilators and reduced hospital and ICU stay. However, interestingly survival at 30-day posthospital admission was lower in the early tracheostomy group, while 90-day post-hospital admission survival was similar in both groups.

Finally, a systematic review of ventilated patients with COVID-19 was done. The early tracheostomy group showed a lower mortality rate than the late tracheostomy with a cut-off of 16.5 days. Interestingly, tracheostomy timing did not influence hospital, ICU stay, and decannulation. It is confirmed that there is no clear impact of the timing of tracheostomy on patient outcomes [20].

## Conclusions

COVID-19 has remained a significant global challenge, prompting the development of new therapeutic approaches and reevaluating existing ones. In this study, tracheostomy in ventilated COVID-19 patients assessed and investigated the incidence of bacterial infections associated with early versus late tracheostomy. The evidence concerning the optimal timing of tracheostomy is inconclusive. Early tracheostomy might improve outcomes, such as decreased ventilator-associated pneumonia and shorter hospital stays. However, the decisions to do so should be tailored to each patient, considering their specific clinical situation alongside the potential risks and benefits of the procedure. Results showed no significant association between the timing of tracheostomy and the incidence of ventilator-associated pneumonia. Yet, it was observed that different bacterial strains influence patient mortality. Consequently, further research on this critical topic is recommended to address the findings and overcome the limitations observed in the current study.
